# Knowledge, perception and usage of E-learning among medical undergraduates in Andhra Pradesh, India

**DOI:** 10.6026/973206300200196

**Published:** 2024-02-29

**Authors:** Roza Bhaisare, Shahana Syed, Gaurav Rangari

**Affiliations:** 1 Department of Community Medicine, Nimra Institute of Medical sciences, Vijayawada, Andhra Pradesh, India; 2 Department of Pharmacology, AIIMS Mangalagiri, Andhra Pradesh, India

**Keywords:** E-learning, medical undergraduates, perceptions, knowledge

## Abstract

Electronic gadgets help to get study material outside classroom and it is used for self-directed learning which helps user to overcome limitations of Conventional teaching methods. Medical education is constantly growing and evolving with rapid speed. So, it is necessary to keep the upcoming medical graduates and established medical practitioners updated in this competitive world. For this, E-learning is the important tool in the medical field. This cross-sectional study was conducted in the medical college of Andhra Pradesh. Total 285 medical students were included in this study and data is obtained from semi structured self-administrative questionnaire. Among 285 students 99.6% students had smartphone and 89.5% were aware of E-learning. Most of the students 96.1% were Conversant with use of internet and 75.4% participants were aware of academic websites. Majority of students 73% agreed that E-learning helps in writing exams and 87.4% students recommended provision of free internet by institute for e-learning. All students were aware about e-learning and using it in the medical field. So, it is necessary to provide essential facility at institutional level for e-learning.

## Background:

In educational system, use of Internet has become important technological tool, it influences positively in the field of education [1-2]. Amongst students of medical field, computers assisted learning has become popular [3-4]. E-learning is a new method of teaching and learning using internet, computers, electronic media and online resources [5- 6]. It is also called distributed learning, web-based learning, Internet-based learning, online-learning and computer-assisted learning [7]. If we compare with the last decade, the use of computer and internet-based learning in developing countries are growing now [8]. Blended learning is traditional teaching combined with e-learning i.e. demonstration is followed by an online tutorial [9]. It is new term in education but its concept familiar to most educators and it keeps the upcoming doctors and established physicians updated in this competitive world [10-11]. Therefore, it is of interest to document data on knowledge, perception and usage of E-learning among medical undergraduates in Andhra Pradesh, India.

## Materials and Methods:

## Study area:

Nimra Institute of Medical Sciences, Vijayawada, Andhra Pradesh.

## Study population:

MBBS students studying in 1st and 3rd year (no 2nd year batch during study period)

## Study design:

ross sectional descriptive study

## Study period:

2 months from June 2021 to July 2021

## Study sample:

285 medical undergraduates was included in this study

## Study tool:

A semi structured self-administrative questionnaire was given to every student who was willing to participate in this study.

## Ethics Committee Approval:

The Ethical approval was taken from the Institutional Ethics Committee of Nimra Institute of Medical Sciences, Vijayawada, Andhra Pradesh for conducting this study.

## Methodology:

285 medical students were included by convenient sampling method. A Semi structured self-administrative questionnaire is prepared in English. An informed consent was taken before collecting the data by explaining the purpose of the study. Questionnaires were given to the students in person and under supervision of investigator data was collected. Data was entered into Microsoft Excel and all entries were cross checked against with the questionnaire.

## Statistical analysis:

The data obtained from excel sheet was analyzed by using SPSS V28.

## Results:

**Figure 1:**
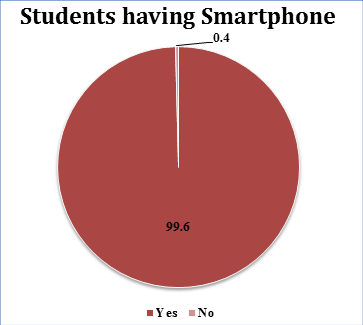
Students having Smartphone (n=285) (Original)

**Figure 2:**
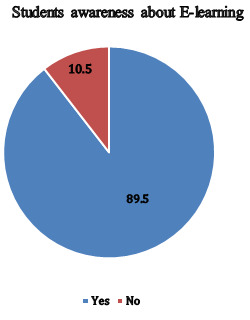
Students awareness about E-learning (n=285) (Original)

**Figure 3:**
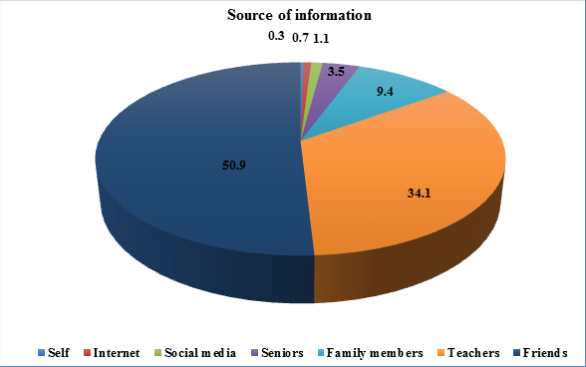
Source of information about awareness of E-learning (n=255) (Original)

**Figure 4:**
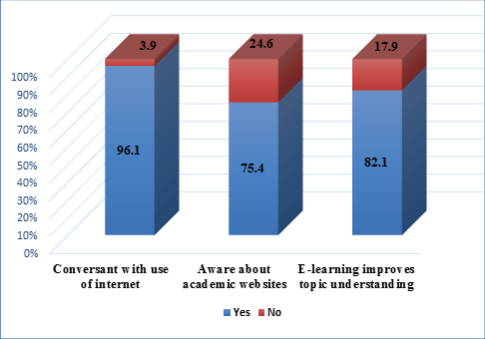
Students thinking about e-learning (n=285) (Original)

**Figure 5:**
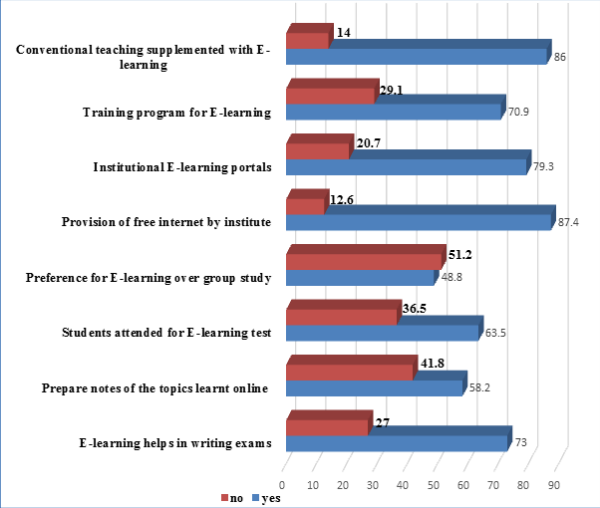
Students recommendation and usage of e-learning (n=285) (Original)

**Figure 6:**
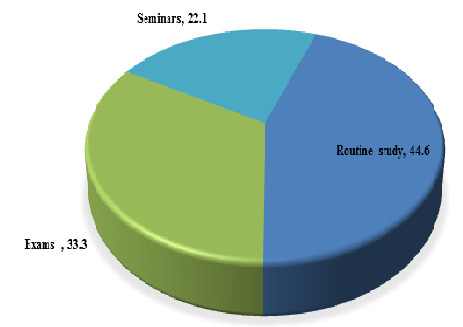
Helpfulness of E-learning in academic (n=285) (Original)

**Figure 7:**
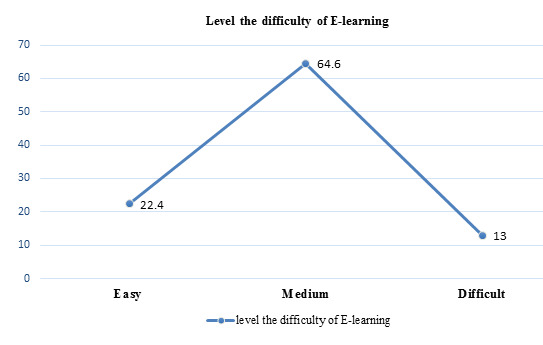
Students about the level the difficulty of E-learning (n=285) (Original)

**Figure 8:**
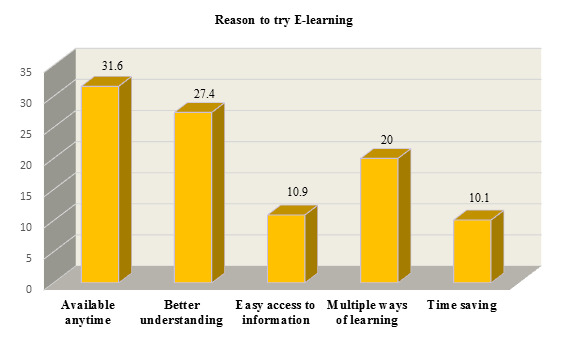
Reason that students tried E-learning (n=285) (Original)

A total 285 students were included with mean age 20.61 and SD 1.572. The Socio demographic information was shown in Table 1. (Table 1) In this study, almost equal participation of 1st year and 3rd year MBBS students presents i.e. 51.6% participants from 3rd year MBBS and 48.4% participants from 1st year MBBS. Most of the participants were Female 65.6% (187) and male participants were 34.4% (98). Maximum students 56.1% (160) were hostellers whereas 43.9% (125) students were day scholars. Most of the student's 61.4% (175) belong to upper class family with per capita income above Rs.7008, followed by students 20.0% (57) upper middle class family with per capita income between Rs.3504 and 7007, 15.1% (43) participants middle class family with per capita income between Rs.2102 and 3503 and 1.8% (5) with per capita income of ranging Rs.1051-2101 and 1.8%(5) with rs.1050 and below. More than three fourth students 78.9% (225) were from urban area and 21.1% (60) students were from rural area. Almost all students 99.6% (284) had Smartphone. (Figure 1) More than half 53% (151) students had laptop and 47% (134) didn't have laptop. Most of the students 89.5% (255) were aware of E-learning (Figure 2). Out of 255 students who are aware of E-learning, friends 50.9 % (130) being the main source of the information (Figure 3). Maximum students 96.1% (274) were Conversant with use of internet and only 3.9% (11) weren't conversant. About three fourth 75.4% (215) participants were aware of academic websites while 24.6% (70) weren't aware. In this study, maximum students 82.1% (234) think E-learning improves topic understanding and 17.9% (51) doesn't agree with improvement of topic understanding using E-learning (Figure 4). More than half of the participants 71.9% (205) spend more time on social sites than with E-learning and 28.1% (80) doesn't spend more time on social sites. More students 29.5% (84) spent time for less than 2hours browsing online every day, then 20.7% (59) students for less than 3 hours ,17.5% (50) students for less than 4 hours followed by 15.8% (45) students for less than 1 hour and 16.5% (47) students for more than 4 hours. Students 28.4% (81) spending less than 2 hours' time on browsing social sites while 17.9 % (51) students spent more than 4hours. (Table 2) Maximum students 83.5% (238) browse for study videos or animations while only 16.5% (47) don't browse for study videos. More than half students 73% (208) agree with E-learning helps in writing exams. Almost equal students, 58.2% (166) students make the notes of the topic learnt online. More than half of the students 63.5% (181) took E-learning test. Around48.8% (139) students chose to prefer E-learning over group study while 51.2% (146) students prefer group study. Maximum students 87.4% (249) recommended provision of free internet by institute. While 79.3% (226) students recommended for institutional E-learning portals. Most of the students 70.9 % (202) think training program is required for E-learning. Whereas 86% (245) participants recommended conventional teaching supplemented with E-learning (Figure 5). 44.6% (127) students agreed that E-learning is most helpful during routine study, 33.3% (95) students during or in exams while 22.1% (63) think it for seminar (Figure 6). More than half of the students 64.6% (184) felt the medium level difficulty, 22.4% (64) students felt E-learning is easy, 13% (37) students felt E-learning is difficult (Figure 7). 31.6% (90) students tried E-learning as its available anytime, 27.4% (78) tried for better understanding, 20%(57) as multiple ways of learning using E-learning, followed by 10.9%(31) students tried E-learning because of easy access to information and 10.1%(29) students tried E-learning as time saving (Figure 8).

## Discussion:

Knowledge regarding online-learning methods, perceptions about e-learning and usage of e-learning in medical studies is of interest. 1st year MBBS (48.4%) and 3rd year MBBS (51.6 %) students were included in this study. However, the study conducted by Hiwarkar *et al.* and Visalam *et al.* included 1st year MBBS students [12- 13]. Abbasi *et al.* conducted the survey on MBBS (53.4%) and BDS (46.6%) students [14]. Also, study of Omprakash *et al.* included MBBS (53.4%) and BDS (46.6%) students [15]. Majority of the participants were female 65.6% while male participants were 34.4%. Similar finding was observed in study of Visalam *et al.*, Abbasi *et al.* and Omprakash *et al.* In this study, maximum students 56.1% were hostellers and 43.9% students were day scholars, this finding is similar to the finding of study conducted by Visalam *et al.* Almost all students 99.6% had Smartphone. This observation is similar with the study conducted by Hiwarkar *et al.* Whereas more than fifty percent (53%) of students had laptop, this is in contrast of study of Hiwarkar *et al.*, where only few students (20%) had laptop [12]. Most of the students (89.5%) were aware of E-learning and (10.5 %) weren't aware of E-learning. The friends (50.9%) being the major source of the information, followed by teachers (34.1%), family members (9.4%), senior's students (3.5%) and social media (1.1%). The current study observed that maximum students (96.1%) were Conversant with use of internet and around three fourth (75.4%) participants were aware of academic websites. Similar finding was observed in study of Hiwarkar M *et al.* where almost all students (93.22%) were conversant with use of internet and 77.97 % students were aware of academic websites. Maximum students (82.1%) agreed that E-learning improves topic understanding. The study of Hiwarkar *et al.* also showed similar findings where 90.68% students accepted that e-learning improves topic understanding. About three fourth of the students (71.9%) spend more time on social sites than with E-learning. This finding is in contrast with study of study of Hiwarkar M *et al.* where around half of the students spend more time on social sites than with E-learning [12]. More students 29.5% were spending time less than 2 hours for browsing online every day, followed by 20.7% students spending less than 3 hours, 17.5% students spending less than 4 hours, 15.8% students spending less than 1 hour and 16.5% students spending time more than 4 hours for browsing online every day. Hiwarkar *et al.* showed that 35% students were spending time up to 2 hours every day whereas 32% students less than one hour. Most of the students 56.5% were spending 1-2-hour time daily for browsing social sites. Majority of the students 83.5% browse for study videos or animations related to subjects. This observation is similar with the study of Hiwarkar M *et al.* where 91.1% students browse for study videos or animations. More than half of the students (73%) agreed that E-learning helps in writing exams where other 27% students didn't agree. A study of Hiwarkar *et al.* also observed that 64.83% students agreed that e-learning helps in writing exams. Around 58.2% students make the notes of the topic learnt online and 41.8% students don't make notes. The observation is similar to the study of Hiwarkar *et al.* (50.42%). More than half of the students 63.5% took e-learning test while 36.5% students didn't. This finding is in contrast to the study of Hiwarkar *et al.*, where only 27.97% students took e-learning test. 48.8% students chose to prefer e-learning over group study while around 51.2% students prefer group study over e-learning. In study of Hiwarkar *et al.*, 54.66% students prefer e-learning over study group. Maximum students (87.4%) recommended provision of free internet by institute and 79.3% students recommended for institutional E-learning portals. Most of the students (70.9%) think training program is required for E-learning. 86% students recommended conventional teaching should be supplemented with E-learning. In study conducted by Hiwarkar *et al.* also observed that 90.68% students recommended provision of free internet by institute, 93.64 % students recommended institutional E-learning portals, 72.03% % students think training program is required for E-learning and 84.32% students recommended conventional teaching should be supplemented with E-learning [12]. Also study of Omprakash *et al.* showed similar observation that 84% students agreed that e-learning should be used as supplementary tool [15]. 44.6% students agreed that E-learning is most helpful during routine study, 33.3% students agreed that it was helpful during or in exams while 22.1% students agreed that it was helpful for seminar. Around similar finding was observed in study of Hiwarkar *et al.*, 48.3% students agreed e-learning helpful in Routine study, 35.8% students agreed that it was helpful in exam and 16% students accepted that e-learning was helpful in Seminar [12]. 22.4% students felt E-learning was easy, more than half of the students 64.6% felt the medium level of difficulty during e-learning and 13% students felt E-learning is more difficult. This finding was contrast with study of Hiwarkar *et al.*, 42% students felt e-learning Easy, 51.3 % students felt medium and students felt Difficult 6.8% [12]. In this study, 31.6% students tried E-learning because it was available at any time, 27.4% students tried e-learning for better understanding, 20% students tried e-learning as multiple ways of learning. Whereas 10.9% students tried E-learning because of easy access to information and 10.1% students tried E-learning because it is time saving.

## Conclusion:

About three fourth of the students agreed that e-learning was helpful in writing exam and it improves subject understanding. Also, half of the participants preferred e-learning over group study. It was also found that more than half students felt medium level of difficulty during e-learning. Hence, e-learning should be supplemented with traditional method of teaching. Also, the institute should arrange training program for students related to e-learning. The free provision of internet in the campus should be provided by the institute.

## Figures and Tables

**Table 1:** Socio demographic variables (n= 285) (Original)

**Category**	**Variable**	**Count**	**%**
Sex	Female	187	65.60%
	Male	98	34.40%
MBBS year	1st year	137	48.40%
	3rd year	146	51.60%
Day scholar / Hosteller	Day scholar	125	43.90%
	Hosteller	160	56.10%
Permanent Residence	Rural area	60	21.10%
	urban area	225	78.90%
Per capita income of family	7008 and above	175	61.40%
	3504-7007	57	20.00%
	2102-3503	43	15.10%
	1051-2101	5	1.80%
	below 1050	5	1.80%

**Table 2:** Students spending time everyday browsing social sites (n=285) (Original)

**Time spent**	**Frequency**	**Percentage**
<1hour	80	28.1
<2hours	81	28.4
<3hours	39	13.7
<4hours	34	11.9
>4hours	51	17.9
